# Exosomes from miR‐20b‐3p‐overexpressing stromal cells ameliorate calcium oxalate deposition in rat kidney

**DOI:** 10.1111/jcmm.14555

**Published:** 2019-09-05

**Authors:** Jing Shi, Junyao Duan, Huijie Gong, Yuewen Pang, Ling Wang, Yongji Yan

**Affiliations:** ^1^ Department of Urology, Dongzhimen Hospital Beijing University of Chinese Medicine Beijing China

**Keywords:** calcium oxalate deposition, exosomes, microRNAs, miR‐20b‐3p, stromal cells

## Abstract

Hyperoxaluria‐induced calcium oxalate (CaOx) deposition is the key factor in kidney stone formation, for which adipose‐derived stromal cells (ADSCs) have been used as a therapeutic treatment. Studies revealed that miR‐20b‐3p is down‐regulated in hypercalciuric stone‐forming rat kidney. To investigate whether ADSC‐derived miR‐20b‐3p‐enriched exosomes protect against kidney stones, an ethylene glycol (EG)‐induced hyperoxaluria rat model and an in vitro model of oxalate‐induced NRK‐52E cells were established to explore the protective mechanism of miR‐20b‐3p. The results showed that miR‐20b‐3p levels were decreased following hyperoxaluria in the urine of patients and in kidney tissues from animal models. Furthermore, treatment with miR‐20b‐3p‐enriched exosomes from ADSCs protected EG‐induced hyperoxaluria rats, and cell experiments confirmed that co‐culture with miR‐20b‐3p‐enriched exosomes alleviated oxalate‐induced cell autophagy and the inflammatory response by inhibiting ATG7 and TLR4. In conclusion, ADSC‐derived miR‐20b‐3p‐enriched exosomes protected against kidney stones by suppressing autophagy and inflammatory responses.

## 1 INTRODUCTION

Kidney stones are a common and frequently occurring ailment that seriously affects human health. They are related to many factors including genetics, environment and metabolism. At present, the mechanisms of pathogenesis remain unclear. There are many types of kidney stones, of which calcium oxalate (CaOx) is the most common component.^1^, ^2^ Therefore, studying the pathogenesis of CaOx‐induced kidney stones and exploring prevention and treatment strategies are important topics in the field of clinical urology research.^3^, ^4^ Many studies have reported that transplantation of mesenchymal stem cells (MSCs) can have therapeutic effects on various kidney diseases, due to their potential to differentiate and multiply.^5^, ^6^, ^7^ However, shortcomings of treatments with MSCs cannot be ignored, as inflammation and anoxic microenvironments can result in a high rate of apoptosis and consequently poorer therapeutic effects than expected.^8^ Recent studies have found that only a small proportion of transplanted MSCs survive in host tissues, and the protective role of MSCs in a kidney injury model is related to a paracrine mechanism. MSCs may reduce damage and promote repair by transmitting key signals to cells that survive injury, including secretion of cytokines via paracrine or endocrine mechanisms that alter cell behaviour to exert anti‐inflammatory and anti‐apoptotic effects, and promote angiogenesis.^9^

Recent research found that cells can also communicate with each other by secreting exosomes that are ordinarily stored in intraluminal vesicles and released when the vesicles fuse with the cell wall.^10^ Exosomes can interact with target cells in a variety of ways to mediate the transmission of intercellular information and participate in a variety of physiological and pathological processes. For example, exosomes can enter target cells through membrane receptors or endocytosis, and transmit biological information through mRNAs, microRNAs (miRNAs) and proteins.^11^, ^12^ Mesenchymal adipose‐derived stromal cells (ADSCs) are abundant and are easy to culture in vitro^13^, ^14^ Compared with other mesenchymal SCs, ADSCs have the advantages of convenient acquisition, facile cell harvesting and ease of culture in vitro. Exosomes from ADSCs can avoid endosomal‐lysosomal degradation, unlike polymeric nanoparticles and liposomes, and they lack immunogenicity. These characteristics make exosomes ideal for gene‐drug delivery.^15^ A previously reported high‐throughput microarray study revealed that rno‐miR‐20b‐3p is down‐regulated in hypercalciuric stone‐forming rat kidney tissue compared with normal tissue, but the specific regulatory mechanism is unknown.^16^ Multiple studies have confirmed that miRNA‐mediated autophagy and modulation of inflammation play an important role in kidney disease progression.^17^, ^18^, ^19^, ^20^ Bioinformatics analysis (http://www.targetscan.org/) found that rno‐miR‐20b‐3p targets toll‐like receptor 4 (TLR4) and autophagy‐related gene 7 (Atg7), suggesting that the expression of miR‐20b‐3p plays an important role in the regulation of autophagy and inflammation.

In the present study, to clarify the potential role of miR‐20b‐3p in treatment with ADSC‐derived exosomes, we investigated the level of miR‐20b‐3p following hyperoxaluria in animal kidney stones models. We investigated the target genes of miR‐20b‐3p and the relationships between miR‐20b‐3p, inflammation and autophagy, both in vivo and in vitro. This research indicates that exosomes derived from miR‐20b‐3p‐overexpressing ADSCs achieve greater treatment effects on kidney stones.

## 2 MATERIALS AND METHODS

### 2.1 Animals

All Sprague Dawley (SD) rats (male, weight 180‐220 g) were purchased from Shanghai Sippr‐Bk Laboratory Animals and maintained under controlled conditions with a 12/12 hours light/dark cycle, a temperature of 22 ± 3°C and humidity of 60 ± 5%. All animals could acquire food and water freely and were treated according to the Guide for the Care and Use of Laboratory Animals. All rats were anesthetized by intraperitoneal injection of sodium pentobarbital (30 mg/kg) before surgical procedures. All experiments were approved according to the Ethics Committee of Beijing University of Traditional Chinese Medicine.

### 2.2 Culturing ADSCs

Adipose tissues obtained from killed SD rats (male, weight 180‐230 g) were washed with phosphate‐buffered saline (PBS) and cut into pieces (1 × 1 mm). After digestion with collagenase, tissues were centrifuged at 4000 × *g* for 5 minutes. The resultant cell pellet was then suspended in Dulbecco's modified Eagle's medium (DMEM) containing 10% foetal bovine serum (FBS), 1% penicillin‐streptomycin, 2 mmol/L L‐glutamine and cultured in a controlled atmosphere with 5% CO_2_ for 48 hours at 38°C. Cells were removed and added to fresh culture medium that was subsequently changed every 3 days. When cells were ~90% confluent, they were passaged and used at passage three. Cells were incubated with conjugated monoclonal antibodies against CD29, CD44, CD90, CD105, CD31 and CD45 to confirm the identity of ADSCs, while isotype‐identical antibodies (PharMingen) served as controls. ADSCs were then fixed in 1% paraformaldehyde, and a FACSCalibur flow cytometer (BD Biosciences) and FlowJo software (FlowJo) were used for quantitative analyses. Logarithmic fluorescence intensities were recorded for 10 000‐20 000 cells per sample.

### 2.3 Isolation and analysis of exosomes

The negative control (NC) and miR‐20b‐3p mimics were provided by GenePharma and transfected into ADSCs at a final concentration of 20 nmol/L using Lipofectamine 3000 (Invitrogen Life Technologies). ADSCs were collected for analysis of miR‐20b‐3p expression at 48 hours after transfection.

Adipose‐derived stromal cells (miR‐20b‐3p overexpression, control and NC groups) at 80%‐90% confluence were washed with PBS and cultured in microvascular endothelial cell growth medium‐2 media deprived of FBS. We then used 1 × serum replacement solution (PeproTech) to supplement ADSCs for 24 hours. To remove dead cells and debris, ADSCs were centrifuged at 300 × *g* for 10 minutes and 2000 × *g* for 10 minutes, after which 5 mL of ExoQuick‐TC reagent (System Biosciences) was mixed with 10 mL of supernatant. After centrifugation at 1500 × *g* for 30 minutes, the exosome‐containing pellet was resuspended in nuclease‐free water. TRIzol‐LS (Invitrogen) and an Exosomal Protein Extraction kit (Invitrogen) were used for extracting total RNA and protein, respectively. Exosomes were used immediately for experiments or stored at −180°C. A NanoSight LM10 (Malvern Instruments) nanoparticle tracking system was used to determine the sizes of purified exosomes.

Western blotting was used to measure CD63 and TSG101 protein levels. To assess the concentration of proteins in exosomes, we used a bicinchoninic acid assay kit (Beyotime). Transmission electron microscopy (TEM) using a Libra 120 instrument (Zeiss) was performed to analyse the ultrastructure of the vesicles.

### 2.4 Patient samples

We assessed 30 kidney stone patients admitted to the Dongzhimen Hospital, Beijing, China. These patients were clinically diagnosed as having kidney stones according to hospital records. Thirty healthy controls were also recruited from the Dongzhimen Hospital, Beijing, China. Urine samples were taken within 24 hours of symptom onset and frozen in liquid nitrogen. Ethical approval for the study was provided by the Independent Ethics Committee of Dongzhimen Hospital, Beijing, China, affiliated with the Beijing University of Traditional Chinese Medicine. Informed and written consent was obtained from all patients or their advisors according to Ethics Committee guidelines.

### 2.5 Hyperoxaluria rat model

Thirty‐two male SD rats were randomly divided into control, ethylene glycol (EG), EG + exosomes (Exo) and EG + Exo‐miR‐20b‐3p groups (n = 8). Control group rats had free access to tap water. The hyperoxaluria rat model was induced by allowing rats in the EG group free access to drinking water containing 1% EG. We isolated exosomes (400 μg of protein) in 200 μL PBS and administered them by intravenous injection daily for 4 weeks for Exo groups, whereas control rats received an equal volume of normal saline.

### 2.6 Kidney histological observation

Mouse kidney tissue samples were fixed in 10% PBS‐formalin for at least 24 hours and embedded in paraffin for histological assessment. Samples were sectioned (5 μm) and stained with haematoxylin and eosin (H&E) using standard protocols. They were then examined microscopically for structural changes and observed under a light microscope (Olympus) to evaluate kidney damage.

### 2.7 Immunohistochemistry

Samples were deparaffinized with xylol, sliced into 4 µm sections and rehydrated using a graded ethanol series. A heat‐induced epitope protocol was used for antigen retrieval (95°C for 40 minutes). Samples were incubated in methanol containing 0.3% hydrogen peroxide to block endogenous peroxidases. Samples were blocked with protein serum using a Vectastain Elite ABC kit (Vector Laboratories, Inc) then incubated overnight at 4°C with polyclonal rabbit anti‐human TLR4 and ATG7 antibodies (Cell Signaling Technology) at 1:1000 dilutions. After washing three times in TBST (150 mmol/L NaCl, 10 mmol/L TRIS‐HCl, pH 7.6), sections were incubated with secondary antibody for 20 minutes at room temperature. Peroxidase‐conjugated biotin‐streptavidin complex (Dako) was then applied to sections for 20 minutes. Sections were visualized with 3,3’‐diaminobenzidine and counterstained with haematoxylin. For the NC, non‐immune serum was used instead of primary antibody.

### 2.8 Renal function detection

For urine collection, one day before euthanasia, the rats were housed in metabolic cages for urine collection. The 24 h urine volumes were collected. For serum collection, anaesthetized rats were killed and blood was withdrawn. Blood samples were kept at room temperature for 2 hours. Serum was collected after centrifugation at 840 g for 15 minutes. Urine BUN, creatinine, NGAL and serum creatinine were analysed using commercial kits purchased from Nanjing Jiancheng Bioengineering Institute, according to manufacturers’ protocols.

### 2.9 Cell culture

NRK‐52E cells were purchased from the American Type Culture Collection (ATCC) and cultured in DMEM supplemented with 10% FBS. NRK‐52E cells were transfected with miR‐20b‐3p mimic, TLR4 and ATG7 overexpression vectors for 48 hours (95% air and 5% CO_2_) and cultured with or without 0.75 mmol/L oxalate for 48 hours. All cellular experiments were independently performed in triplicate.

### 2.10 Cell transfection

For miR‐20b‐3p overexpression, the miR‐20b‐3p mimic or corresponding NC (mimic NC) was purchased from GenePharma. ADSC was transfected with either the miR‐20b‐3p mimic or mimic NC at a final concentration of 50 nmol/L using Lipofectamine 2000 (Invitrogen) according to the manufacturer's protocol. The cells were then used for miR‐20b‐3p expression analyses or for other experiments after 48 hours of transfection. To assess ATG7 and TLR4 expression, ATG7 and TLR4 overexpression vector or NC (vector) were constructed by GenePharma. NRK‐52E cells were transfected with the ATG7 and TLR4 overexpression vector at a final concentration of 50 nmol/L, using Lipofectamine 2000 reagent according to manufacturer's protocols. Cells were used for ATG7 and TLR4 expression analysis or other experiments after 48 hours of transfection.

### 2.11 Cell viability assay

Cells were placed in 96‐well plates at an initial density of 5000 cells per well, and 500 μg/mL MTT was added and incubated for 4 hours. The resulting blue formazan was dissolved in 10% sodium dodecyl sulphate (SDS), 5% isobutanol, 0.01 mol/L HCl and all plates were scanned by a microplate reader (Thermo Scientific) at 570 nm with 630 nm as a reference. Cell viability was normalized as the percentage relative to controls.

### 2.12 Western blotting

Membranes were incubated with primary antibodies recognizing TLR4 (1:1000), ATG7 (1:1000), Phospho‐p65 (1:1000), Phospho‐IκB (1:1000), p65 (1:1000), IκB (1:1000), LC3B (1:100), p62 (1:1000) and β‐actin (1:1000) (Cell Signaling Technology) after non‐specific binding was blocked. Enhanced chemiluminescence detection (Abcam) was used for visualizing immunoreactive proteins. ImageJ 1.44 was used for analysing immunoreactive labelling.

### 2.13 mRNA extraction and RT‐PCR analysis

TRIzol was used to isolate total RNA from urine, ADSCs, exosomes, NRK‐52E cells and kidney tissues, and cDNA was synthesized from 1 μg total RNA using SuperScript reverse transcriptase and oligo dT18 primers. Taq DNA polymerase (TaKaRa Bio) was used with 1 μL of first‐strand cDNA amplification product as template for standard PCR with 30 cycles at 94°C for 30 seconds, 55°C for 30 seconds and 72°C for 30 seconds. Relative expression levels were calculated using the 2^−ΔΔCT^ method.

### 2.14 Enzyme‐linked immunosorbent assay

Enzyme‐linked immunosorbent assay (ELISA) kits were purchased from RapidBio. The concentration of inflammatory factors IL‐6, IL‐1β and TNF‐α in cell supernatants or serum were estimated using an ELISA kit according to the manufacturers’ procedures.

### 2.15 Crystal cell adhesion assay

After receiving the appropriate treatments, NRK‐52E cells were cultured in a 6‐well plate until they reached 100% confluence. After washing with PBS, cells were incubated in DMEM containing 40 µg/mL Ponceau S‐labelled COM crystals for 10 minutes at 37°C in a humidified atmosphere of 5% CO_2_, then washed vigorously with PBS three times to remove unbound COM crystals. Finally, images were captured under an IX‐71 bright field inverted microscope (Olympus), and the number of adherent (or remaining) crystals labelled by Ponceau S in at least 10 randomized high‐power fields per was were counted.

### 2.16 Autophagic flux analysis

NRK‐52E cells were transfected with mRFP‐GFP‐LC3 for 24 hours. After transfection, mRFP‐GFP‐LC3‐NRK‐52E cells were fixed with 4% paraformaldehyde and stained with 10 μmol/L Hoechst 33 342. Cell images were obtained using an Operetta High Content Imaging System (Perkin‐Elmer) and analysed using Harmony analysis software (Perkin‐Elmer). Cells were detected using green fluorescent protein (GFP) or monomeric red fluorescent protein (mRFP). Puncta in autophagosome and autolysosomes were stained yellow and red, respectively, in merged images. Autophagic flux was determined by the increased percentage of red puncta in merged images.

### 2.17 Electron microscopy

Cells were fixed with 2.5% glutaraldehyde in PBS and stored at 4°C until embedding. Cells were post‐fixed with 1% osmium tetroxide followed by an increasing gradient dehydration step using ethanol and acetone. Cells were then embedded in Araldite, and ultrathin sections were obtained (50‐60 nm), placed on uncoated copper grids and stained with 3% lead citrate‐uranyl acetate. Images were examined using a CM‐120 electron microscope (Philips).

### 2.18 Statistical analysis

SPSS 21.0 software was used to carry out one‐way analysis of variance (anova) and *t* tests among groups. Data are expressed as mean ± standard deviation (SD), and differences were considered significant at *P* < .05.

## 3 RESULTS

### 3.1 Treatment with ADSC‐derived miR‐20b‐3p‐enriched exosomes suppresses EG‐induced kidney CaOx crystal deposition in vivo

**Figure 1:**
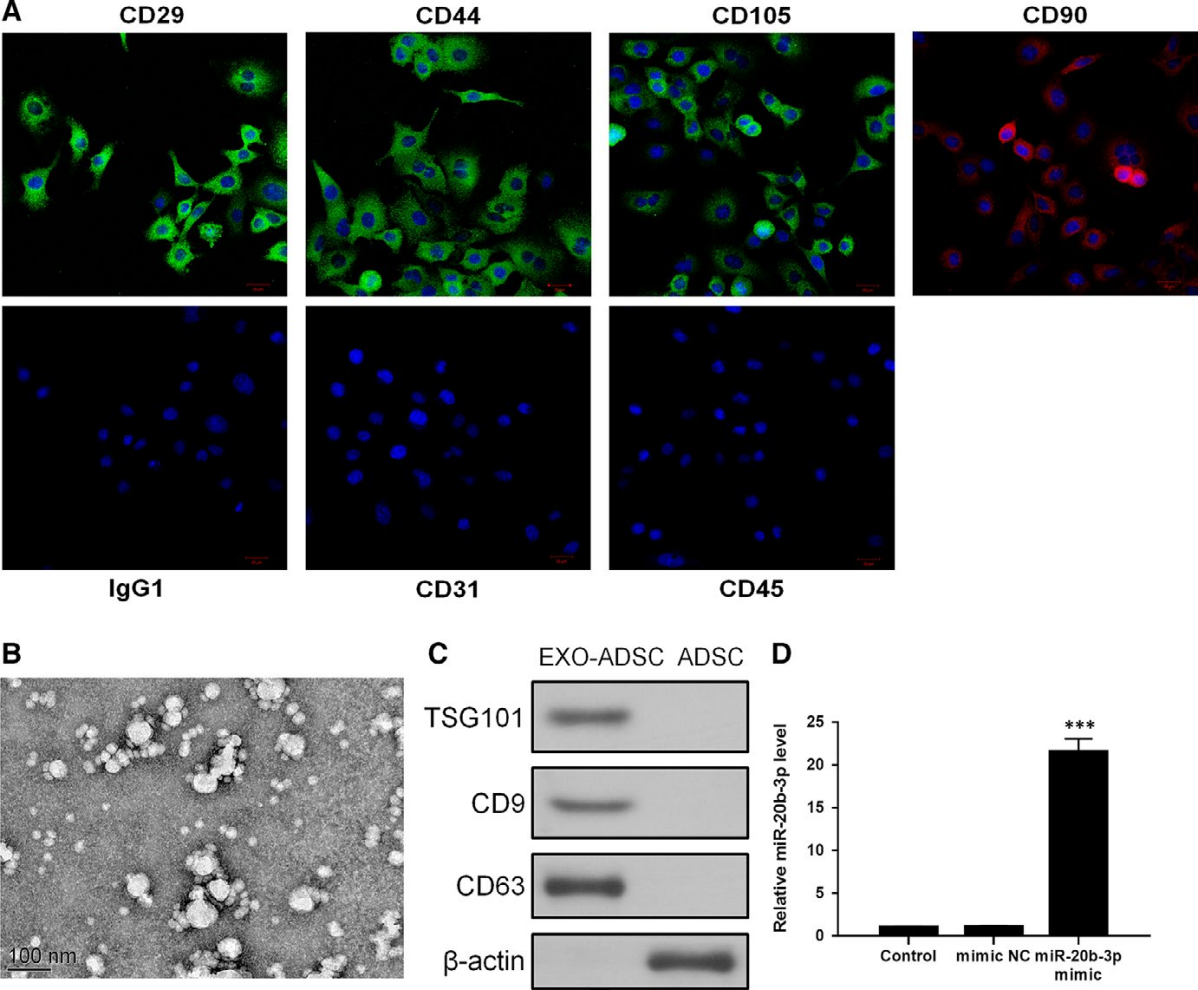
Identification of adipose‐derived stromal cells (ADSCs) and exosomes. A, Assessment of cell surface markers by immunofluorescence staining. ADSCs were positive for mesenchymal stem cell markers CD29, CD44, CD90 and CD105, but negative for endothelial markers CD31 and CD45. Negative isotype controls are shown. Scale bars = 100 mm. B, Transmission electron microscopy image of exosomes. C, Western blots using CD63 and TSG101 as markers of ADSC‐derived exosomes. D, Reverse transcription PCR detection showing expression of miR‐20b‐3p in exosomes after transfection. Data are presented as the mean ± SD (n = 3). ***P < .001 vs control group

First, we used cell surface markers to detect ADSC cell phenotypes. The results showed that ADSCs were positive for MSC markers CD29, CD44, CD90 and CD105, but were negative for endothelial markers CD31 and CD45 (Figure 1). TEM analyses showed that exosomes purified from ADSCs were 30‐100 nm in diameter (Figure 1B). Western blotting confirmed the expression of exosome markers CD63 and TSG101 (Figure 1C). In previous research, miR‐20b‐3p was shown to be down‐regulated in hypercalciuric stone‐forming rat kidney compared to normal tissue.^16^ We transfected ADSCs with miR‐20b‐3p mimic for 48 hours and cultured with serum‐free medium. The RT‐PCR results showed that the level of miR‐20b‐3p in exosomes was increased after transfection with miR‐20b‐3p mimic (Figure 1D).

**Figure 2:**
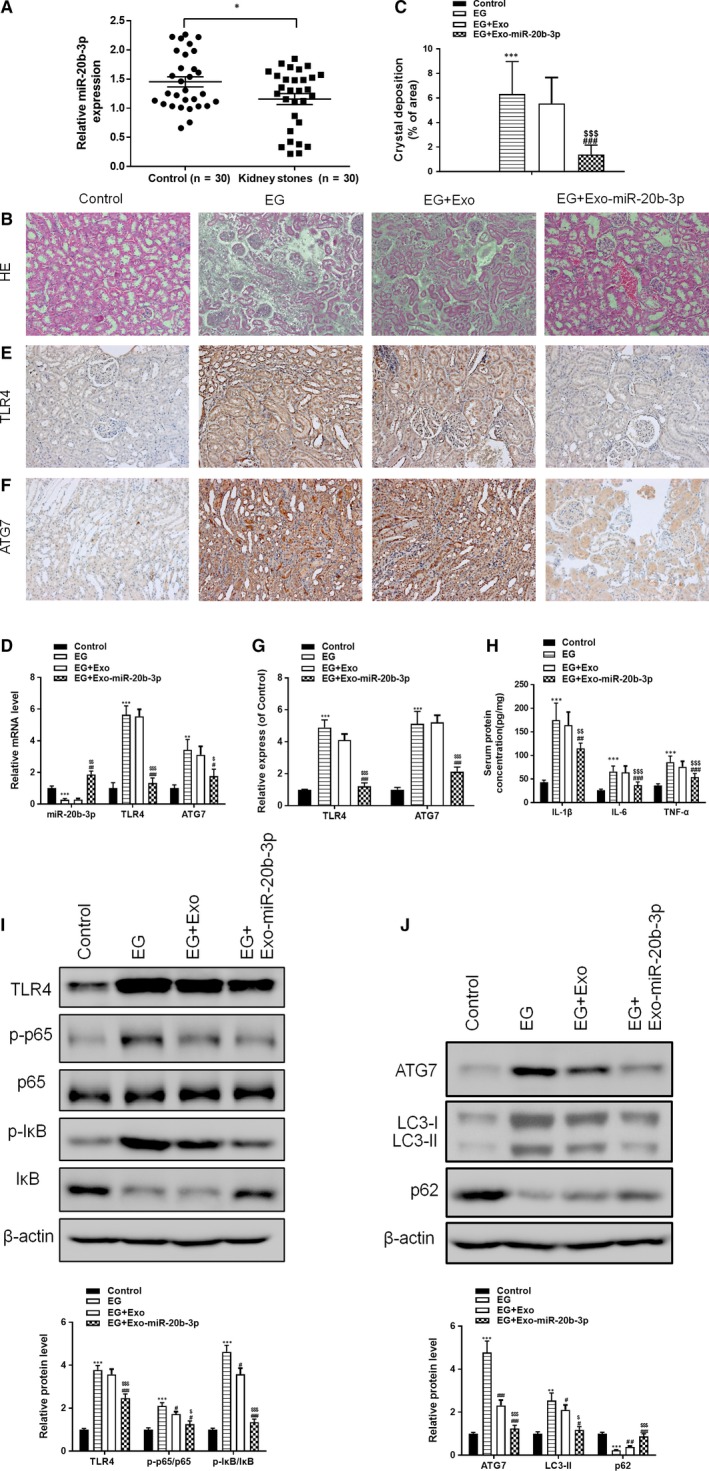
Treatment with adipose‐derived stromal cell‐derived miR‐20b‐3p‐enriched exosomes alleviates ethylene glycol (EG)‐induced kidney CaOx crystal deposition in rats. (A) RT‐PCR analyses of miR‐20b‐3p levels in the urine of kidney stone patients. (B and C) Photomicrographs of kidney sections from rats obtained under dark field illumination with polarized light. Retained crystals exhibit strong birefringence (200× magnification). (D) Levels of miR‐20b‐3p, ATG7 and TLR4 determined by RT‐PCR. Levels of ATG7 (E) and TLR4 (F) in kidney tissue were assessed by immunohistochemical staining and quantified (G). (H) Rat serum protein concentrations for IL‐1β, IL‐6 and TNF‐α determined by ELISA. (I) Inflammation‐related proteins TLR4, p65 and IkB determined by Western blotting and quantified. (J) Autophagy‐related proteins Atg7, P62 and LC3 determined by Western blotting and quantified. Data are presented as the mean ± SD (n = 6). **P < .01 and ***P < .001 vs control group; #*P* < .05, ##*P* < .01 and ###*P* < .001 vs EG group; $*P* < .05, $$*P* < .01 and $$$*P* < .001 vs EG + Exo group

To investigate the functions of miR‐20b‐3p in kidney stones, we collected urine from 30 kidney stone patients and 30 healthy controls. Reverse transcription PCR (RT‐PCR) analyses demonstrated that expression of miR‐20b‐3p was significantly decreased in the urine of kidney stone patients compared with the control group (Figure 2A). Next, we used an EG‐induced hyperoxaluria rat model to evaluate the effects of exosomes on CaOx crystal deposition in vivo*.* The results indicated that EG‐induced hyperoxaluria in the kidney was significantly decreased in rats treated with miR‐20b‐3p‐enriched exosomes (Figure 2B,C). Furthermore, we measured the release of urinary renal injury markers creatinine, BUN and NGAL. As shown in Table 1, serum creatinine and urinary creatinine, BUN and NGAL levels in the EG group were increased significantly compared with controls and significantly decreased after treatment with miR‐20b‐3p‐enriched exosomes. To determine whether the therapeutic effects of miR‐20b‐3p‐enriched exosomes were related to autophagy and inflammation, we used rat kidney tissue for RT‐PCR and Western blot analysis. RT‐PCR results showed that the expression of miR‐20b‐3p was decreased after EG treatment. Following treatment with miR‐20b‐3p‐enriched exosomes, levels of miR‐20b‐3p were increased, which indicates that miR‐20b‐3p could be delivered by exosomes into the kidney (Figure 2D). RT‐PCR (Figure 2D) and immunohistochemistry assay (Figure 2E‐G) showed that levels of TLR4 and ATG7 were significantly increased in EG‐induced hyperoxaluria rat kidneys compared with controls, while miR‐20b‐3p‐enriched exosome treatment reversed this increase. Western blot results indicated that the TLR4/NF‐κB pathway was involved in EG‐induced hyperoxaluria. Following treatment with miR‐20b‐3p‐enriched exosomes, EG‐induced TLR4 expression and p65/IκB phosphorylation were significantly suppressed (Figure 2I). ELISA results revealed that miR‐20b‐3p‐enriched exosomes reversed the increase in serum inflammatory‐related protein concentrations of IL‐1β, IL‐6 and TNF‐α in rats (Figure 2H). Further Western blot results showed that miR‐20b‐3p‐enriched exosome treatment had a significant effect on regulating autophagy inhibition, which increased the expression of LC3‐II/I and Atg7 and decreased p62 expression in kidney samples (Figure 2J).

**Table 1:** Parameters for ethylene glycol (EG)‐induced rats in urine or serum (n = 6 in each group)

Group	Parameters			
	Urine BUN (mg/dL)	Urine creatinine (mg/dL)	Urine NGAL (mg/dL)	Serum creatinine (mg/dL)
Control	10.47 ± 1.32	8.97 ± 1.22	0.18 ± 0.014	0.133 ± 0.035
EG	53.7 ± 6.54***	37.8 ± 5.21***	1.78 ± 0.21***	0.763 ± 0.114***
EG + Exo	54.8 ± 6.23	35.8 ± 5.07	1.76 ± 0.18	0.712 ± 0.089
EG + Exo‐miR‐20b‐3p	22.7 ± 3.22###, $$$	18.7 ± 2.58##, $$	0.87 ± 0.064###, $$$	0.278 ± 0.032###, $$$

*** *P* < .001 vs control group.

^##^ *P* < .01.

^###^ *P* < .001 vs EG group.

^$$^ *P* < .01.

^$$$^ *P* < .001 vs EG + Exo group.

### 3.2 Co‐culturing with miR‐20b‐3p‐enriched exosomes alleviated oxalate‐induced injury in NRK‐52E cells by inhibiting autophagy via ATG7

**Figure 3:**
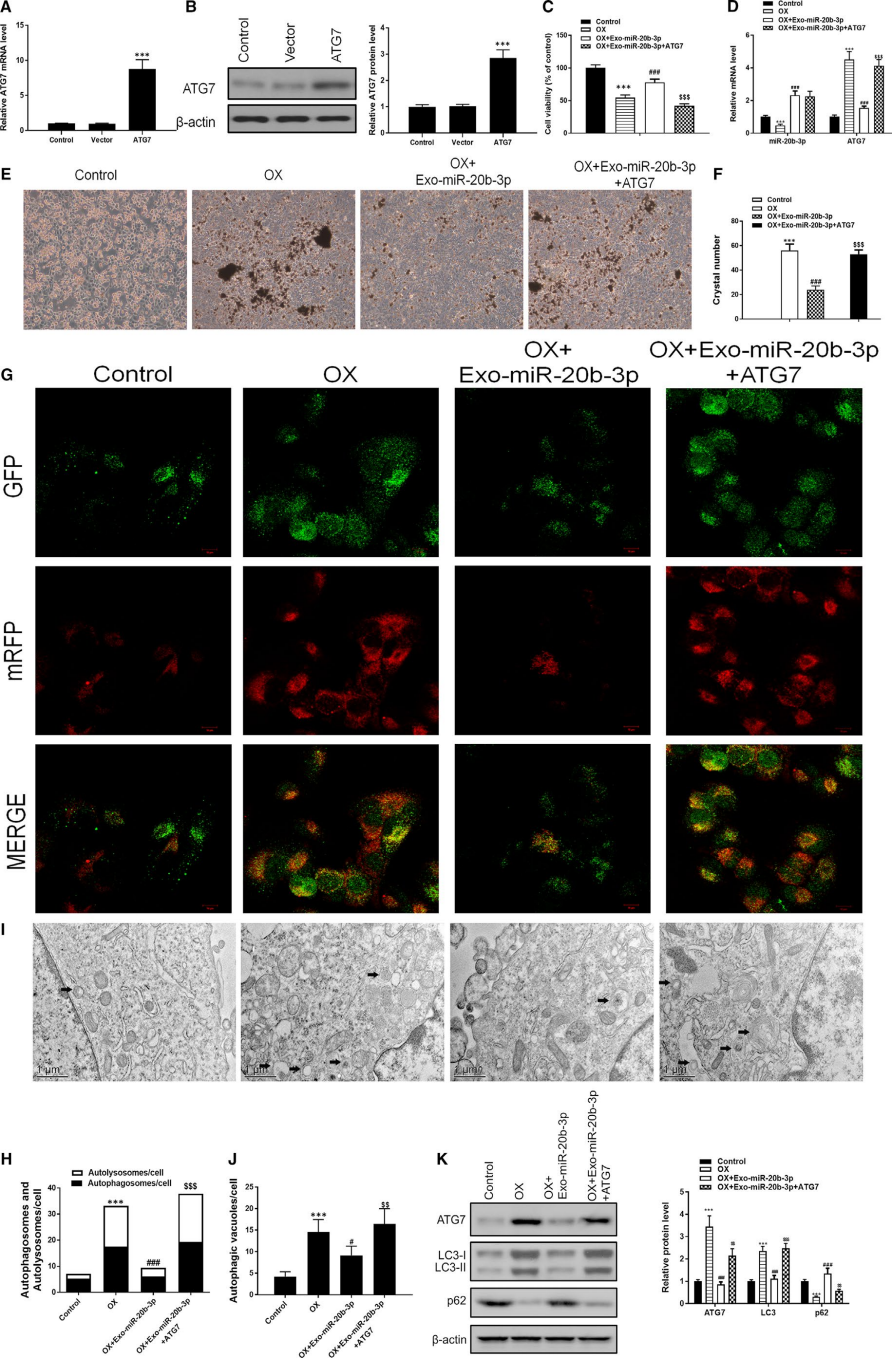
Co‐culturing with miR‐20b‐3p‐enriched exosomes alleviates oxalate‐induced injury in NRK‐52E cells by inhibiting autophagy via ATG7. A and B, ATG7 levels in NRK‐52E cells after ATG7 overexpression for 48 h determined by RT‐PCR and Western blotting. C, Cell viability assessed by MTT assay. D, Levels of miR‐20b‐3p and ATG7 determined by RT‐PCR. (E and F) Crystal cell adhesion assay determination and quantification. G and H, Representative images of immunofluorescence staining of mRFP‐GFP‐LC3 in NRK‐52E cells. Representative profiles of autophagosomes (RFP + GFP+dots) and autolysosomes (RFP + GFP‐dots) per cell section assessed by confocal microscopy are shown and were quantified. I and J, Autophagic vacuoles (autophagosomes) were detected by transmission electron microscopy (TEM). Representative TEM images are shown and typical autophagosomes are marked with black arrows. The number of autophagosomes per cell was calculated by counting the number of double‐membrane organelles in 10 cells. K, Autophagy‐related proteins Atg7, P62 and LC3 determined by Western blotting and quantified. Data are presented as the mean ± SD (n = 3). **P < .01 and ***P < .001 vs control group; #*P* < .05, ##*P* < .01 and ###*P* < .001 vs OX group; $*P* < .05, $$*P* < .01 and $$$*P* < .001 vs OX + Exo‐miR‐20b‐3p group

An ATG7 overexpression vector was constructed and transfected into NRK‐52E rat renal tubular epithelial cells for 48 hours before detection by RT‐PCR and Western blotting (Figure 3A,B) to investigate the mechanism of ATG7 in OX‐induced cell autophagy. RT‐PCR results showed that oxalate decreased miR‐20b‐3p and increased ATG7 levels, consistent with the in vivo results. NRK‐52E cells were then stimulated with oxalate (0.75 mmol/L) for 48 hours, and MTT results showed that OX‐induced inhibition of cell viability was significantly alleviated by miR‐20b‐3p‐enriched exosomes, whereas cell viability was decreased after ATG7 overexpression (Figure 3C). After co‐culturing with miR‐20b‐3p‐enriched exosomes, miR‐20b‐3p was increased and ATG7 was decreased, demonstrating the inhibition effect of miR‐20b‐3p on ATG7 (Figure 3D). Next, we examined the protective effects of miR‐20b‐3p on crystal adhesion. The results showed that miR‐20b‐3p‐enriched exosomes clearly suppressed adhesion of CaOx crystals induced by OX in NRK‐52E cells, and ATG7 overexpression significantly increased adhesion (Figure 3E,F). Immunofluorescence staining and confocal microscopy were used to determine the levels of autolysosomes and autophagosomes in NRK‐52E cells (Figure 3G). Levels of lysosomes and phagosomes were decreased after co‐culture with miR‐20b‐3p‐enriched exosomes (Figure 3H). TEM analysis of autophagic vacuoles confirmed these results (Figure 3I). The number of autophagic vacuoles was diminished in cells co‐cultured with miR‐20b‐3p‐enriched exosomes and increased after overexpression of ATG7 (Figure 3J). Western blotting showed that LC3 conversion was reduced and levels of p62 were increased when OX‐induced NRK‐52E cells were co‐cultured with miR‐20b‐3p‐enriched exosomes, indicating that autophagy induced by oxalate was suppressed, while ATG7 overexpression reversed this inhibition by miR‐20b‐3p (Figure 3K). Overall, these results indicate that miR‐20b‐3p‐enriched exosomes inhibit OX‐induced autophagy in NRK‐52E cells.

### 3.3 Co‐culturing with miR‐20b‐3p‐enriched exosomes alleviates oxalate‐induced injury in NRK‐52E cells by inhibiting inflammatory responses via TLR4

**Figure 4:**
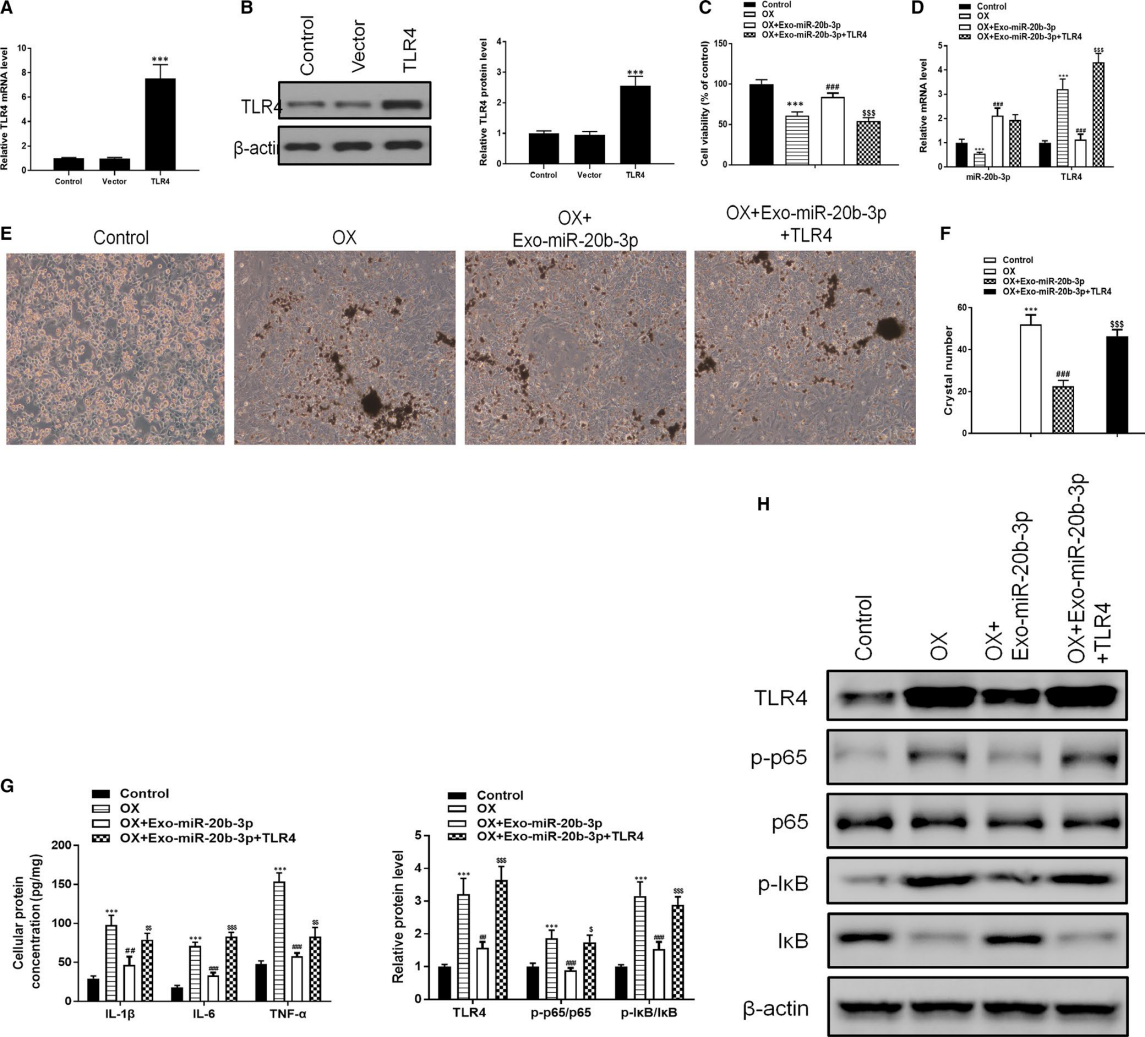
Co‐culturing with miR‐20b‐3p‐enriched exosomes alleviates oxalate‐induced injury in NRK‐52E cells by inhibiting inflammatory responses via TLR4. A and B, TLR4 levels in NRK‐52E cells after TLR4 overexpression for 48 h determined by RT‐PCR and Western blotting. C, Cell viability assessed by MTT assay. D, Levels of miR‐20b‐3p and ATG7 determined by RT‐PCR. E and F, Crystal cell adhesion assay determination and quantitation. G, Cellular protein concentrations for IL‐1β, IL‐6 and TNF‐α determined by ELISA. H, Inflammation‐related proteins TLR4, p65 and IkB determined by Western blotting and quantified. Data are presented as the mean ± SD (n = 3). **P < .01 and ***P < .001 vs control group; #*P* < .05, ##*P* < .01 and ###*P* < .001 vs OX group; $*P* < .05, $$*P* < .01 and $$$*P* < .001 vs OX + Exo‐miR‐20b‐3p group

To further analyse the mechanism of the TRL4 pathway in inflammatory responses, we constructed a TLR4 overexpression vector and transfected it into NRK‐52E cells for 48 hours before assessing by Western blotting and RT‐PCR (Figure 4A,B). The results showed that OX‐induced inhibition of cell viability was significantly reversed by miR‐20b‐3p‐enriched exosomes, whereas cell viability was decreased after TLR4 overexpression (Figure 4C). RT‐PCR results showed that oxalate increased the level of TLR, whereas co‐culturing with miR‐20b‐3p‐enriched exosomes decreased the level TRL4 (Figure 4D). Crystal adhesion results showed that TLR4 overexpression significantly reversed the suppressive effect of miR‐20b‐3p (Figure 4E,F). ELISA results showed that cellular protein concentrations for IL‐6, IL‐1β and TNF‐α induced by oxalate were suppressed by miR‐20b‐3p‐enriched exosomes, whereas this effect was reversed by TLR4 overexpression (Figure 4G). Western blotting results showed that miR‐20b‐3p‐enriched exosomes inhibited OX‐induced phosphorylation of p65 and IκB, whereas TLR4 overexpression reversed the inhibitory effect of miR‐20b‐3p (Figure 4H).

### 3.4 Both ATG7 and TLR4 are targets of miR‐20b‐3p

**Figure 5:**
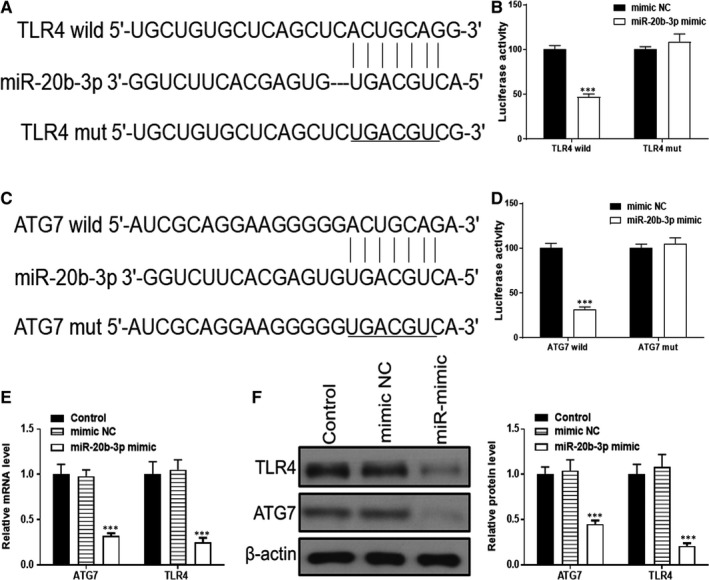
Both ATG7 and TLR4 are targets of miR‐20b‐3p. A and B, Complementary sequences between miR‐20b‐3p and the 3′‐untranslated region (UTR) of wild‐type or mutated version of ATG7 and TLR4 were obtained using publicly available algorithms. C and D, The 3′‐UTR of ATG7 and TLR4 was fused to the luciferase coding region and co‐transfected into NRK‐52E cells with miR‐20b‐3p mimic to confirm that miR‐20b‐3p is a target of miR‐20b‐3p. Relative luciferase activity was determined at 48 h after transfection. E and F, Levels of TLR4 and ATG7 determined by Western blotting and RT‐PCR. Data are presented as the mean ± SD (n = 3). ***P < .001 vs mimic NC

ATG7 and TLR4 were identified as potential targets of miR‐20b‐3p (http://www.targetscan.org/). To investigate the relationships between miR‐20b‐3p, ATG7 and TLR4, we searched for putative miR‐20b‐3p binding sites in ATG7 and TLR4 (Figure 5A,B), and generated luciferase reporter constructs in which these putative binding sites were mutated. Mutant (mut) and wild‐type (wt) luciferase reporter constructs were transfected into NRK‐52E cells together with miR‐20b‐3p mimics or negative control (miR‐NC). Interestingly, miR‐20b‐3p mimics significantly inhibited luciferase activity in NRK‐52E cells transfected with wt constructs, but luciferase activity was not affected in cells transfected with mut constructs (Figure 5C,D), indicating that miR‐20b‐3p directly targets ATG7 and TLR4. RT‐PCR and Western blotting results proved that miR‐20b‐3p overexpression significantly suppressed ATG7 and TLR4 expression at both protein and mRNA levels in NRK‐52E cells (Figure 5E,F).

## 4 DISCUSSION

Kidney stones are one of the most commonly occurring urological disorders worldwide. According to one study, ~12% of men and 5% of women in the US will suffer from kidney stones during their life‐time.^21^ Approximately 80% of kidney stones are CaOx, and the recurrence rate of CaOx‐based stones can be as high as 60 to 80%.^22^ Exosomes are vesicles containing a diversity of macromolecular substances including proteins, mRNAs, miRNAs and long non‐coding RNAs (LncRNAs) that have the ability to regulate intracellular signalling pathways.^23^ Previous research showed that ADSC transplantation was an effective but relatively inefficient treatment.^24^, ^25^ The purpose of the present study was to find ways to enhance the treatment efficacy using ADSC‐derived exosomes to treat kidney stones.

Our results showed that the level of miR‐20b‐3p was significantly decreased in the serum of kidney stone patients. To test whether this was responsible for the ameliorating effects of exosomes on kidney stones, we used SD rats as an EG‐induced kidney stone model. Our findings indicated that treatment with miR‐20b‐3p‐containing exosomes significantly decreased CaOx crystal deposition and renal cell injury in vitro and in vivo by suppressing oxidative stress and autophagy.

The function of autophagy is to ensure the delivery of metabolic substrates to renal tissue to fulfil their energy demand during stress, especially in an inflammatory environment, thereby supporting cell survival.^26^, ^27^ However, overactive autophagy may lead to autophagic cell death, known to contribute to kidney injury development, but its role in mediating renal cell damage remains a controversial subject.^28^ Some studies indicated that autophagy activation prevents renal tubular epithelial cells against damage induced by nephrotoxic drugs.^29^ On the other hand, other studies found that an increase in autophagic activation leads to renal tubular epithelial cell death in ischaemia‐reperfusion or tunicamycin‐induced kidney injury.^30^ Accordingly, autophagy activation has dual defensive and harmful effects on renal tubular epithelial cells. Previous kidney stone research found that inhibition of autophagy attuned oxidative injury of the mitochondrial membrane and ameliorated calcium crystal deposition.^31^, ^32^, ^33^ Furthermore, increasing evidence implies a potential connection between autophagy and inflammatory responses. For example, inhibition of autophagy by 3‐MA or small interfering RNA (siRNA) knockdown of BECN1 attenuates CaOx crystal‐induced renal tubular epithelial cell injury by suppressing reactive oxygen species (ROS) and inflammation.^31^, ^34^ Additionally, the NFκB pathway can be activated by oxalate in renal tubular cells, and inhibition of NFκB can effectively reduce crystal adhesion.^35^, ^36^ In our current study, we found elevated levels of autophagy markers in renal tissues in the EG group, and miR‐20b‐3p‐containing exosomes suppressed EG‐ and OX‐induced autophagy by targeting ATG7, which indicates that the effects of miR‐20b‐3p are related to autophagy blockage. Follow‐up dual‐luciferase reporter assays revealed that TLR4 is a direct target of miR‐20b‐3p. In vitro experiments showed that miR‐20b‐3p inhibited inflammatory responses by suppressing the level of TLR4, a protein that makes an important contribution to regulating inflammatory responses in kidney injury.^37^, ^38^ Our study revealed that miR‐20b‐3p inhibited OX‐induced inflammatory factor expression by targeting TLR4. These results indicate that the protective effects of miR‐20b‐3p are related to TLR4 suppression.

Collectively, this research showed that treatment with ADSC‐derived miR‐20b‐3p‐enriched exosomes had an effective therapeutic effect on EG‐induced kidney stones in rats. We found that miR‐20b‐3p targeted ATG7‐mediated autophagy and TLR4‐mediated inflammatory responses. These findings provide new insight into the therapeutic effects and molecular mechanisms of ADSC‐derived miR‐20b‐3p‐enriched exosomes in treating kidney stones.

## CONFLICT OF INTEREST

All authors declare no conflicts of interest.

## AUTHOR CONTRIBUTIONS

YYJ conceived and designed the research; SJ carried out experiments and analysed the data; DJ, GH, PY and WL participated in statistical analyses and data interpretation; SJ drafted and revised the manuscript; all authors approved the final manuscript.
